# Acknowledging the Peer Reviewers of *Journal of Medical Radiation Sciences*, October 2025–September 2026

**DOI:** 10.1002/jmrs.70141

**Published:** 2026-11-05

The editorial review board, the Australian Society of Medical Imaging and Radiation Therapy (ASMIRT) and the New Zealand Society of Medical Imaging and Radiation Therapy (NZSMIRT) acknowledge the following peer reviewers for their dedication and commitment to the journal. Thank you for sharing your valuable time and knowledge.

## Outstanding Reviewers (Contributed Five Reviews or More)

James Stanley

Sibusiso Mdletshe

Edel Doyle

Andrew Kilgour

Cameron Brown

Paul Lockwood

Georgia Halkett

Nicole Zientara

Tracey Pieterse

## The Following Reviewers Contributed Four Reviews

Karen Dobeli

Lynne Hazell

Stephen Knight

## The Following Reviewers Contributed Three Reviews

Dana Jackson

Heather Lawrence

Hesta Friedrich‐Nel

Johnathan Hewis

Luke Barclay

Riaan van de Venter

Scott Jones

Teresa Poon

Tracy Parker

Vicki Braithwaite

## The Following Reviewers Contributed Two Reviews

Adrienne Young

Ahmed Jibril Abdi

Andrea Roletto

Azlinawati Ali

Bismark Ofori‐manteaw

Christine Esther Albantow

Claire Poole

Clare Singh

Elizabeth Brown

Elizabeth McGahan

Emily Skelton

Gary Denham

Jacky Chen

James McNeil

Jason Arts

Jenna Dean

Jeremy Kilgour

Jillian Clarke

Katelyn Cahill

Katrina O'Keefe

Kim Lewis

Kunthi Pathmaraj

Mark Pinkham

Mark Widdowfield

Martin Ian Kamanda

Meegan Shepherd

Michael Chao

Monchai Phonlakrai

Nick Woznitza

Noraniza Azmi

Norhayati Mohd Zain

Peter Kench

Sarah Dowthwaite

Shantel Lewis

Tania Poroa

Toni Kelly

William Antwi

Zhonghua Sun

## The Following Reviewers Contributed One Review

Adam Steward

Afrooz Najafzadeh Abriz

Aladdin Speelman

Albertina Rusandu

Alexey Kolchev

Alexey Surov

Amanda Punch

Amani Bell

Amy Walker

Ana Grilo

Andrew England

Andrew Firman

Andrew Murphy

Anelyn Chui

Anetta Bolejko

Angelina Piccolo

Anthony Arnold

Antonella Fogliata

Ashesh Ranchod

Belinda Van der merwe

Bernadette Byrne

Bornface Chinene

Briana Farrugia

Brock Lamprecht

Bronwyn Hilder

Brooke Osborne

Carminia Lapuz

Chandra Makanjee

Charles Tran

Chelsie O'connor

Chris Williams

Christian ven Emery

Christopher O'brien

Clare Rainey

Clinton Gould

Courtney Luck

Crispen Chamunyonga

Dana S. al Al‐mousa

David Christie

David Stewart

Duminda Satharasinghe

Dustin Osborne

Eko Hidayanto

Emma Rawlings

Emmanuel Gyan

Eric Pei Ping Pang

Erika Cavanagh

Fraide jr Ganotice

Geofery Luntsi

Gerhardus Koch

Glenn Cahoon

Gordon Mander

Hafsa Essop

Hamed Ghaffari

Haydn Kerr

Heidi Bowmast

Huang Juncheng

Ilona Lavender

Imelda Williams

Jacky Chan

Jacob leonard Ago

Jameen Arm

James Elliott

James Hayes

Jemma Blyth

Jeremy Hoang

Joerg Lehmann

Jonathan loui Portelli

Josie Macfarlane

Julianna Hapi

Kam Lee

Katrina Blyth

Katrina Woodford

Kelly Elsner

Kenton Thompson

Kim Fitzgerald

Kirsten Oataway

Kristie Matthews

Kristie Sweeney

Lindiwe Gumede

Maeve Kearney

Mario Perez

Mark Tacey

Maseeh Uz Zaman

Melissa Rosemary Pillay

Melissa Shields

Michael Pinkawa

Michala Short

Mohamed Abuzaid

Mohammad khairi jahidi Mahazer

Mohd. zamzuri Bin che daud

Mona Fazel Ghaziyani

Natalie Rozanec

Nikos Stogiannos

Nurul Ismail

Paul Kane

Paulina Cegla

Peter O'Reilly

Phuong Dung (Yun) Trieu

Pippa Bresser

Pooja Gohil

Rachael Beldham‐collins

Rebecca Height

Rex de Ryke

Robin Hart

Ruth Pape

Ryan Brown

Sahand Hooshmand

Shelley Bulling

Sheryl Foster

Sukja Yoon

Suresh Sukumar

Thandokuhle Khoza

Tom Steffens

Tristan Reddan

Vanessa Panettieri

Victor Lee

Vikneswary Batumalai

Yu Dai

Yu‐Feng Wang

Zanariah Mohd

## Peer Reviewer Recognition

JMRS reviewers can opt in to have their peer review contribution automatically added on Web of Science Reviewer Recognition Services (formerly Publons).

For more information please go to https://authorservices.wiley.com/Reviewers/journal‐reviewers/recognition‐for‐reviewers/publons.html.

## Peer Reviewer Resources

Are you new to conducting and writing peer reviews? Check out these free peer reviewer guidelines and free self‐directed learning modules.

Wiley Journal Reviewers

https://authorservices.wiley.com/Reviewers/journal‐reviewers/index.html

Wiley Researcher Academy: All you need to know to become an effective peer reviewer

https://www.wileyresearcheracademy.com/p/all‐you‐need‐to‐know‐to‐become‐an‐effective‐peer‐reviewer

Web of Science Academy

https://clarivate.com/webofsciencegroup/solutions/web‐of‐science‐academy/

